# Potential Benefits and Harms of a Peer Support Social Network Service on the Internet for People With Depressive Tendencies: Qualitative Content Analysis and Social Network Analysis

**DOI:** 10.2196/jmir.1142

**Published:** 2009-07-23

**Authors:** Yoshimitsu Takahashi, Chiyoko Uchida, Koichi Miyaki, Michi Sakai, Takuro Shimbo, Takeo Nakayama

**Affiliations:** ^5^Department of Epidemiology and Healthcare ResearchKyoto University School of Public HealthKyotoJapan; ^4^Department of NeurologySchool of MedicineKeio UniversityTokyoJapan; ^3^University Health Center Ibaraki UniversityIbarakiJapan; ^2^Department of Clinical Research and InformaticsResearch InstituteInternational Medical Center of JapanTokyoJapan; ^1^Department of Health InformaticsKyoto University School of Public HealthKyotoJapan

**Keywords:** Social network service (SNS), Internet, depression, peer support, social network, qualitative method, content analysis, mixed methods

## Abstract

**Background:**

Internet peer support groups for depression are becoming popular and could be affected by an increasing number of social network services (SNSs). However, little is known about participant characteristics, social relationships in SNSs, and the reasons for usage. In addition, the effects of SNS participation on people with depression are rather unknown.

**Objective:**

The aim was to explore the potential benefits and harms of an SNS for depression based on a concurrent triangulation design of mixed methods strategy, including qualitative content analysis and social network analysis.

**Methods:**

A cross-sectional Internet survey of participants, which involved the collection of SNS log files and a questionnaire, was conducted in an SNS for people with self-reported depressive tendencies in Japan in 2007. Quantitative data, which included user demographics, depressive state, and assessment of the SNS (positive vs not positive), were statistically analyzed. Descriptive contents of responses to open-ended questions concerning advantages and disadvantages of SNS participation were analyzed using the inductive approach of qualitative content analysis. Contents were organized into codes, concepts, categories, and a storyline based on the grounded theory approach. Social relationships, derived from data of “friends,” were analyzed using social network analysis, in which network measures and the extent of interpersonal association were calculated based on the social network theory. Each analysis and integration of results were performed through a concurrent triangulation design of mixed methods strategy.

**Results:**

There were 105 participants. Median age was 36 years, and 51% (36/71) were male. There were 37 valid respondents; their number of friends and frequency of accessing the SNS were significantly higher than for invalid/nonrespondents (*P* = .008 and *P* = .003). Among respondents, 90% (28/31) were mildly, moderately, or severely depressed. Assessment of the SNS was performed by determining the access frequency of the SNS and the number of friends. Qualitative content analysis indicated that user-selectable peer support could be passive, active, and/or interactive based on anonymity or ease of use, and there was the potential harm of a downward depressive spiral triggered by aggravated psychological burden. Social network analysis revealed that users communicated one-on-one with each other or in small groups (five people or less). A downward depressive spiral was related to friends who were moderately or severely depressed and friends with negative assessment of the SNS.

**Conclusions:**

An SNS for people with depressive tendencies provides various opportunities to obtain support that meets users’ needs. To avoid a downward depressive spiral, we recommend that participants do not use SNSs when they feel that the SNS is not user-selectable, when they get egocentric comments, when friends have a negative assessment of the SNS, or when they have additional psychological burden.

## Introduction

Mental health topics are especially popular on the Internet, and there are high levels of untreated and undiagnosed depression in users of Internet depression communities [1]. Depression is an important global public health issue, and an effective approach to prevention may involve the targeted screening of people with chronic diseases as well as those with social isolation [2-5]. Peer support—providing support based on mutual counseling and the sharing of information and experience—is becoming an increasingly important strategy in health care environments that face shrinking financial and human resources [6,7]. Although many Internet-based support groups have emerged, a systematic review did not find conclusive evidence concerning the health benefits of virtual communities and peer-to-peer online support [8]. The benefits of Internet-based support groups for depression have been assessed in two independent studies [9,10].

We have also witnessed a new revolution in the field of communication through the Internet, called Web 2.0 [11]. Social network services (SNSs), such as MySpace or Facebook, are Internet-based Web 2.0 applications that allow the building of online social networks where individuals can share interests and activities. SNS users have increased in number all over the world. Moreover, SNSs for specific health-related purposes have emerged (eg, for quitting smoking or for people with cancer or a mental health disorder). A few studies have examined the risks of MySpace for adolescents [12-15]. However, no SNS-related studies on depression or peer support existed in PubMed as of February 2009. SNSs have thus far been used without knowledge of their benefits and harms.

These social backgrounds have prompted the hypothesis that SNS users with depressive tendencies may wish to seek out their peers and information on depression from their peers. Inherent in this, however, are the detrimental effects of Internet addiction [16] and psychological distress [17]. Some recent studies have shown that obesity, smoking, and happiness can spread through social networks in what is termed a “network phenomenon” [18-20]. This may suggest that negative effects of SNSs can be exaggerated by Internet social networks. Therefore, we sought to address the following questions: (1) What are the characteristics of SNS participants? (2) What kinds of social relationships are there in SNSs? and (3) Why and how do participants use SNSs? These questions are both quantitative and qualitative. Hence, we quantitatively examined characteristics and social relationships and qualitatively examined reasons for usage and the process of SNS usage. Finally, this study aimed to explore the potential benefits and harms of SNSs for people with depressive tendencies in Japan through a concurrent triangulation design of mixed methods strategy (combining quantitative and qualitative methods) and to produce converging findings from both methods [21-24].

## Methods

### Study Setting

**Figure 1:**
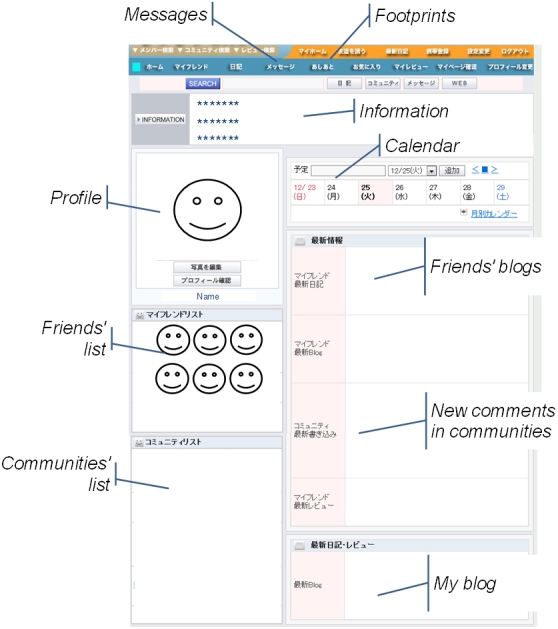
Screenshot of the SNS home page for people with depressive tendencies (Friends: online friends whose profiles are featured as links on one’s own profile. Communities: online communities for people with similar interests or activities. Messages: online messages, such as Web-based email. Information: displays information from the administrator. Invitation function: users who want to participate need an invitation from a participant for registration. Footprints function: participants can ascertain and access the history of another participant. Privacy controls function: participants can choose who can view their profile or contact them.)

An SNS was launched by an individual in April 2006 for people in Japan with self-reported depressive tendencies (Figure 1). Registrants in the SNS described themselves as (1) people who were / had been depressed, (2) people who had family or a friend who was depressed, (3) people who had concerns about depression, or (4) psychiatrists or clinical psychotherapists. They had voluntarily registered in the SNS, which was free of charge and had standard SNS functions. Generally, SNSs in Japan have the following functions: an “invitation” function, a “footprints” function, a “privacy controls” function, and a function to access the SNS from cell phones.

### Study Design and Participants

The study was an observational cross-sectional study in which we conducted two different surveys. In the first survey, the administrator extracted SNS log files, which are electronic records from the SNS database. In the second survey, an Internet questionnaire survey of participants was conducted during March and April 2007. After developing a Secure Sockets Layer compatible website and pretesting for usability and technical functionality, unique IDs and passwords were individually sent to participants through “messages” in the SNS (a nonopen, password-protected survey). Research methods have been presented in compliance with the checklist for reporting results of Internet e-surveys (CHERRIES) [25].

We addressed potential sources of bias. Given that participants were SNS users, we tried to contact them only through the SNS to avoid causing changes in the participants’ behavior. In order to evaluate the reliability of the Internet survey, we compared responses on the questionnaire to data from SNS log files regarding age, gender, and frequency of access. Participants were required to list their diagnoses and medications, and these data were confirmed by a psychiatrist (C Uchida).

### Measures

SNS log files included access logs and data in “profiles,” “blogs,” “communities,” and “friends” (see Figure 1). “Friends” included data of the participant’s network of “friends” (see the Glossary in Multimedia Appendix 1). The questionnaire for the Internet survey included a total of 67 items related to characteristics, activities, and outcomes of four Web pages. Items related to characteristics included age, gender, time of Internet use, job status, whether or not the person lived alone, diagnosis of mood disorders, and assessment of mood states by the Zung Self-Rating Depression Scale and a numerical rating scale. The depression scale ranged from 20 (no depression) to 80 (major depression) and was categorized as follows: not depressed (≤ 39), mildly depressed (40-47), moderately depressed (48-55), and severely depressed (≥ 56) [26,27]. Mood states were measured by a numerical scale that ranged from 0 (good mood) to 10 (bad mood) [28] in the following three situations: (1) during normal time (not using the SNS or Internet), (2) while using the Internet, and (3) while using the SNS. Items related to activities included profile display, frequency of accessing the SNS, frequency of updating blogs (three times or more per week vs less than three times per week), number of communities, and number of friends. Items related to outcomes in the assessment of the SNS were evaluated by the question, “Do you feel your sense of illness management for depression improved compared to before participating in the SNS?” Response choices were “much more,” “more,” “no change,” or “less.” “Much more” and “more” were classified as “positive assessment,” and the other responses were classified as “not positive assessment.” Moreover, the descriptive open-ended question “What are the advantages and disadvantages of participating in the SNS?” was included to explore potential effects of the SNS. Answers to the Internet questionnaire were checked for consistency and saved page by page. All saved data, both complete and incomplete, were analyzed.

### Framework of Analytic Methodology

We used the concurrent triangulation design of mixed methods strategy to analyze both quantitative and qualitative data with qualitative priority [23,24]. First, we examined participant characteristics using statistical analyses (see Step 1 below). Second, we examined reasons for SNS usage and the process of SNS usage with qualitative content analysis [29,30] (see Step 2). Third, we examined the relationships between participants using social network analysis [31-34] (see Step 3). After quantitative and qualitative research questions were examined, these results were integrated based on the mutual validation model, which regards the search for convergent findings deemed to be validity indicators as the most important purpose of triangulation [23]. We explored potential benefits and harms using qualitative results, while we inferred the extent of the benefits and harms using quantitative results. Discrepancies in the results were interpreted and discussed at the discussion session. Multimedia Appendix 2 provides a summary of the framework of analytic methodology.

#### Step 1: Statistical Analyses

To compare variables between groups, we used the Fisher exact test for two categorical variables, Pearson chi-square test for three or more categorical variables, and Student *t* test for continuous variables. The Mann-Whitney U test was used for age, communities, friends, and centrality given that the distribution was estimated to be skewed. Valid respondents, who answered any item in the questionnaire, were compared to invalid/nonrespondents, and people with a “positive” assessment of the SNS were compared to people with a “not positive” assessment. All statistical analyses were performed using SPSS 15.0J (SPSS Inc, Chicago, IL, USA). All *P* values were 2-sided, with *P* < .05 considered statistically significant.

#### Step 2: Qualitative Content Analysis

Descriptive contents of responses to open-ended questions concerning advantages and disadvantages of SNS participation were analyzed using the inductive approach of qualitative content analysis [29,30]. In content analysis, it is assumed that words and phrases that are mentioned often reflect important concerns [35]. However, contents can involve multiple meanings and be latent as well as manifest [29]. In order to achieve trustworthiness [30], contents were inductively organized into codes, concepts, categories, and a storyline based on the grounded theory approach, a commonly used systematic qualitative research method to generate theories regarding social phenomena [21,22,36].

The analysis was performed by multidisciplinary members: a nurse, a pharmacist, and two public health researchers (Y Takahashi and M Sakai). All responses were read and interpreted repeatedly. After discussing the meanings of responses, a coding frame was developed and sentences were coded for analysis. If new codes emerged, the coding frame was changed and sentences were re-read according to the new structure. This constant comparison process was also used to develop concepts, which were then conceptualized into broad categories after further discussion. After discussing the relationship of codes, concepts, and categories, we generated a storyline. We used ATLAS.ti 5.2 (Scientific Software Development GmbH, Berlin, Germany) for data analysis.

#### Step 3: Social Network Analysis

 Data on social relationships among participants were analyzed based on the social network theory [34], in which people were defined as nodes, and relationships were defined as linkages among nodes [31-34]. Social network analysis is the study of social structure that provides a means to quantitatively explore social relationships between people. Commonly used by sociologists, its use in health-related fields is increasing as an effective approach for research centered on describing, exploring, and understanding the relational aspects of health [18-20,32,33]. Network measures and the extent of interpersonal association were calculated based on the theory. The overall social network was graphed with the Kamada-Kawai algorithm using Pajek 1.20 software (University of Ljubljana, Slovenia) from data in “friends” [18-20], according to the sociocentric network approach [31,32]. To identify characteristics of an individual within a network, centrality was measured by (1) degree, (2) closeness, and (3) betweenness [31-33]. These measures of centrality identify the most prominent individual. Degree refers to the sum of individuals who are linked together. Closeness refers to the distance between individuals. Betweenness refers to the number of times an individual connects pairs of others; people with high betweenness centrality are able to play a gatekeeper role, controlling the flow of resources in the network. To examine characteristics of a group, cliques were counted that included three or more individuals connected by all possible connections. These network measures were analyzed using UCINET 6.1 (Analytic Technologies, Lexington, KY, USA).

In order to evaluate the extent of interpersonal association as a whole (whether people with similar attributes tended to be connected with each other or not), we counted numbers of connections sorted by type, including assessment of the SNS (positive vs not positive), depressive state (moderately or severely depressed vs not moderately or severely depressed), and frequency of accessing the SNS (three times or more per week vs less than three times per week). Then we computed odds ratios and 95% confidence intervals. For example, assessment of the SNS was defined as follows: P was a person with a “positive” assessment of the SNS; p was a friend with “positive” assessment; N was a person with “not positive” assessment; n was a friend with “not positive” assessment; p_P was the number of friends with “positive” assessment for people with a “positive” assessment. The odds ratio for assessment of the SNS was computed by (p_P / n_P) / (p_N / n_N).

### Ethical Considerations

We prepared a site that explains the study [37]. We also declared that we collected the SNS log files without personal information and provided an opportunity for refusal (opt-out recruitment). Informed consent was requested from all participants on the first page of the questionnaire (opt-in recruitment). Quantitative data without personal information in SNS log files were collected and analyzed. The questionnaire and SNS log files were anonymized in a linkable fashion. The study protocol was approved by the Ethics Committee of Kyoto University Faculty of Medicine (No. E254, January 12, 2007).

## Results

### Statistical Analyses

**Figure 2:**
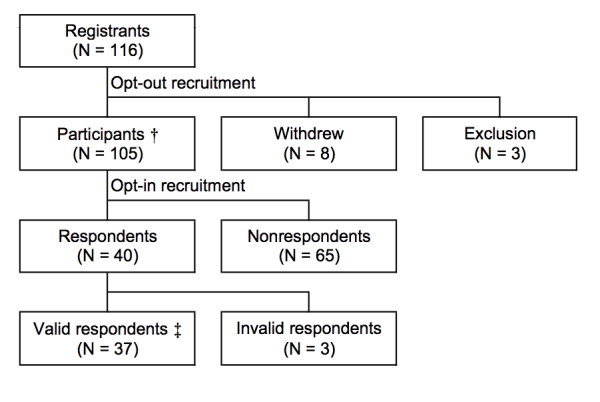
Flow diagram of subjects surveyed and analyzed: “Participants” were subjects of the SNS log file analysis; “Valid respondents” were subjects of the Internet questionnaire analysis; “Withdrew” refers to people who had deleted their accounts themselves; “Exclusion” refers to people who were excluded (one was the administrator, and two had registered within the week before collection of data from the SNS log files); “Respondents” were people who provided informed consent; “Valid respondents” were people who answered any item of the questionnaire; “Invalid respondents” were people who did not answer any item, although they provided informed consent. † is subjects of the SNS log file analysis. ‡ is subjects of the Internet questionnaire survey analysis.

Of the total registrants (N = 116), eight people withdrew. Three people were excluded because one was the administrator and two had registered within the week before collection of data from the SNS log files. Participants (N = 105) were the subjects for the SNS log file analysis. Among the 40 people who responded to the questionnaire, three people were excluded because no item was answered. Valid respondents (N = 37) were the subjects for the questionnaire analysis (Figure 2).

Participants (N = 105) had the following characteristics (Table 1): median age was 36 years (range 21-57), 36/71 (51%) were male, and 47/102 (46%) logged in three times or more per week. Frequency of accessing the SNS by personal computer or cell phone, frequency of updating blogs, and “friends” of valid respondents were significantly higher compared to invalid/nonrespondents (*P* = .003, *P* = .02, and *P* = .008, respectively).

**Table 1:** Characteristics of participants (N = 105)

		n^a^	Total	Response to the Questionnaire		*P*
			(N = 105)	Valid Respondents (N = 37)	Invalid/Nonrespondents^b^ (N = 68)	
**Characteristic**						
Age in years, median (range)		105	36 (21-57)	37 (21-52)	33 (22-57)	.09^c^
Male, n (%)		71	36 (51)	16 (43)	20 (59)	.24^d^
Profile,^e^ n (%)		105	97 (92)	34 (92)	63 (93)	.99^d^
Accessing the SNS,^f^ n (%)						
	By personal computer or cell phone	102	47 (46)	24 (67)	23 (35)	.003^d^
	By cell phone	102	6 (6)	1 (3)	5 (8)	.99^d^
Updating blogs,^f^ n (%)		102	16 (16)	11 (31)	5 (8)	.02^d^
Communities, median (range)		105	3 (2-15)	2 (2-12)	3 (2-15)	.051^c^
Friends, median (range)		105	2 (0-42)	7 (0-27)	2 (0-42)	.008^c^
**Relationship (social network analysis)**						
Centrality, median (range)						
	Degree	105	2 (0-42)	7 (0-27)	2 (0-42)	.008^c^
	Closeness	105	2011 (1925-2177)	1992 (1948-2177)	2011 (1925-2095)	.02^c^
	Betweenness	105	1.1 (0-1718)	27.2 (0-960)	0 (0-1718)	.002^c^



^a^ Several items included missing data.

^b^ Invalid respondents (N = 3); nonrespondents (N = 65).

^c^ Mann-Whitney test.

^d^ Fisher exact test.

^e^ Number of people who had written their profile.

^f^ Three times or more per week.

Table 2 shows characteristics of valid respondents (N = 37). Median age was 37 years (range 21-52), and 16/37 people (43%) were male. Moreover, 32/35 people (91%) could be diagnosed with mood disorders, 28/31 (90%) were mildly, moderately, or severely depressed, and 19/37 (54%) had a “positive” assessment of the SNS. The frequency of accessing the SNS by personal computer or cell phone and “friends” of people with a “positive” assessment of the SNS were significantly higher than for people with a “not positive” assessment (*P* = .02 and *P* = .01, respectively). When comparing the mood state (measured by the numerical scale) while using the SNS and during normal time, people with a “positive” assessment of the SNS showed a greater improvement in their mood than people with a “not positive” assessment (*P* = .07).

**Table 2:** Characteristics and outcomes of valid respondents (N = 37)

		n^a^	Total	Assessment of the SNS^b^				*P*^c^
			(N = 37)	Positive (N = 19)	Not Positive (N = 16)	No Assessment (N = 2)		
**Characteristic**								
Age, median (range)		37	37 (21-52)	37 (26-51)	32 (21-52)	44.5 (40-49)		.30^d^
Male, n (%)		37	16 (43)	8 (42)	7 (44)	1 (50)		.99^e^
Internet use (per week), n		35						
	40+ hours		3	2	1	0		.66^f^
	10-39 hours		19	8	9	2		
	≤ 9 hours		13	8	5	0		
Not working, no (%)		34	16 (47)	11 (58)	5 (33)	0 (0)		.19^e^
Living alone, n (%)		17	5 (29)	2 (18)	3 (50)	0 (0)		.28^e^
Diagnosis, n (%)		35	32 (91)	19 (100)	12 (80)	1 (100)		.08^e^
Medication, n (%)		37	27 (73)	17 (89)	10 (63)	0 (0)		.11^e^
Profile,^g^ n (%)		37	34 (92)	17 (89)	15 (94)	2 (100)		.99^e^
Accessing the SNS,^h^ n (%)								
	By personal computer or cell phone	36	24 (67)	16 (89)	8 (50)	0 (0)		.02^e^
	By cell phone	36	1 (3)	1 (6)	0 (0)	0 (0)		.99^e^
Updating blogs,^h^ n (%)		36	11 (31)	6 (33)	5 (31)	0 (0)		.99^e^
Communities, median (range)		37	2 (2-12)	2 (2-12)	2 (2-6)	3 (2-4)		.71^d^
Friends, median (range)		37	7 (0-27)	8 (1-21)	2 (0-27)	2 (1-3)		.01^d^
**Relationship (social network analysis)**								
Centrality, median (range)								
	Degree	37	7 (0-27)	8 (1-21)	2 (0-27)	2 (1-3)		.01^d^
	Closeness	37	1992 (1948-2177)	1977 (1951-2177)	2008 (1948-2034)	2056 (2034-2078)		.03^d^
	Betweenness	37	27 (0-960)	74 (0-459)	3 (0-960)	0 (0-0)		.02^d^
**Outcome**								
Depressive state,^i^ mean ± SD		31	50.9 ± 9.7	52.8 ± 8.7	50.4 ± 9.0	38.5 ± 19.1	.47^j^	
	Not depressed, n		3	0	2	1	.29^f^	
	Mildly depressed, n		6	5	1	0		
	Moderately depressed, n		12	5	6	1		
	Severely depressed, n		10	6	4	0		
Mood state,^k^ mean ± SD								
	(A) During normal time	31	5.6 ± 2.3	6.2 ± 2.1	5.5 ± 2.1	1.5 ± 2.1	.41^j^	
	(B) While using the Internet	31	4.9 ± 2.4	5.6 ± 2.2	4.7 ± 2.3	1.5 ± 2.1	.31^j^	
	(C) While using the SNS	31	5.1 ± 2.0	5.0 ± 2.0	5.6 ± 1.6	2.0 ± 2.8	.37^j^	
	Difference (B) − (A)	31	−0.7 ± 2.0	−0.6 ± 2.3	−0.8 ± 1.9	0.0 ± 0.0	.78^j^	
	Difference (C) − (A)	31	−0.5 ± 1.9	−1.2 ± 2.3	0.1 ± 1.2	0.5 ± 0.7	.07^j^	


^a^ Several items included missing data.

^b^ Assessment of the SNS was evaluated by the question “Do you feel your sense of illness management for depression improved compared to before participating in the SNS?” Response choices were “much more,” “more,” “no change,” or “less.” “Much more” and “more” were classified as “positive assessment,” and the other responses were classified as “not positive assessment.”

^c^ Comparing positive and not positive assessments of the SNS.

^d^ Mann-Whitney test.

^e^ Fisher exact test.

^f^ Pearson chi-square test.

^g^ Number of people who had written their profile.

^h^ Three times or more per week.

^i^ Depressive states were measured by the Zung Self-Rating Depression Scale and were categorized as follows: not depressed (≤ 39), mildly depressed (40-47), moderately depressed (48-55), and severely depressed (≥ 56).

^j^ Student t test.

^k^ Mood states were measured by the numerical scale, which ranged from 0 (good mood) to 10 (bad mood).

### Qualitative Content Analysis

Potential benefits and harms were examined by qualitative content analysis for 30 valid answers to the open-ended question; 19 concepts and 7 categories were developed (Table 3). A developed concept was described by < >, and a developed category was described by << >>.

Through the analysis, we generated the following storyline that described the reasons and process of SNS usage. The positive comments revealed that some channels (eg, message, blog, and community) or some functions (eg, invitation function, footprints function, and privacy controls function) ensured <<Advantage conditions>> like <Anonymity>, <Easiness>, and <Expectation>, creating a domain where participants could face each other honestly and obtain <<peer support>>. This indicated that the SNS helped network members (1) <Recognize the existence of peers>, who were others suffering from a similar disease; (2) <Acquire information> about their disease, treatment, and experience; (3) <Narrate their experience>; (4) <Support each other> through online interaction; and (5) <Encourage peer support> more quantitatively and/or qualitatively (they made more friends and/or they got closer friends). As an <<Advantage consequence>>, peer support enabled them to understand themselves and <Feel positive>. For some participants, peer support may even have led to <Changing behavior>, such as changing treatment as a consequence of the support.

On the other hand, few participants listed <Egocentric comments> and <Infrequent usage> as <<Disadvantage conditions>> of participation. <Solely cyber communication> with the SNS and intensified <Dependency> by depressed people were identified as negative aspects that encumbered some members with additional psychological burdens. Such increases in psychological burden could subsequently trigger a <Downward depressive spiral>, with the SNS exacerbating certain members’ symptoms, like <Reading negative comments>, <Being depressed>, and <Writing negative comments>. As a <<Disadvantage consequence>>, they experienced <Disappointment> in SNS participation.

**Table 3:** Concepts and categories developed by qualitative content analysis (N = 30)

		Assessment of the SNS (N = 30)^a^		
		Positive (N = 19)	Not Positive (N = 10)	No Assessment (N = 1)
**Advantage Aspect**^**b**^				
<<Advantage conditions>>				
	<Anonymousness>	54	30, 114	
	<Easiness>	95	85	
	<Expectation>	69	85	
<<Peer support>>				
	<Recognizing the existence of peers>	9, 24, 39, 76, 84, 95, 96	19	93
	<Acquiring information>	39, 76, 95	64	
	<Narrating their experiences>	24, 57, 76		
	<Supporting each other>	24, 49, 58, 70, 84, 92, 95, 113	19, 55, 64, 87, 114	
	<Encouraging peer support>	3, 21, 105	64, 106	
<<Advantage consequence>>				
	<Feeling positive>	9, 21, 39, 58, 76, 92	59	93
	<Changing behavior>	49, 76, 113		
**Disadvantage Aspect**				
<<Disadvantage conditions>>				
	<Egocentric comments>	3		
	<Infrequent usage>	69, 96	106, 114	
<<Additional psychological burdens>>				
	<Solely cyber communication>	57, 76	64	
	<Dependency>	21, 24	59, 109	
<<Downward depressive spiral>>^c^				
	<Downward depressive spiral >^c^		87	
	<Reading negative comments>	58	30	
	<Being depressive>	39, 70	109	
	<Writing negative comments>	58		
<<Disadvantage consequence>>				
	<Disappointment>		30	


^a^ Numbers stand for anonymous registrants’ IDs, corresponding to Figure 3.

^b^< > denotes a concept; << >> denotes a category.

^c^ After <Downward depressive spiral> was developed as a concept, a category <<Downward depressive spiral>> was developed that included four concepts: <Downward depressive spiral>, <Reading negative comments>, <Being depressive>, and <Writing negative comments>.

### Social Network Analysis

**Figure 3:**
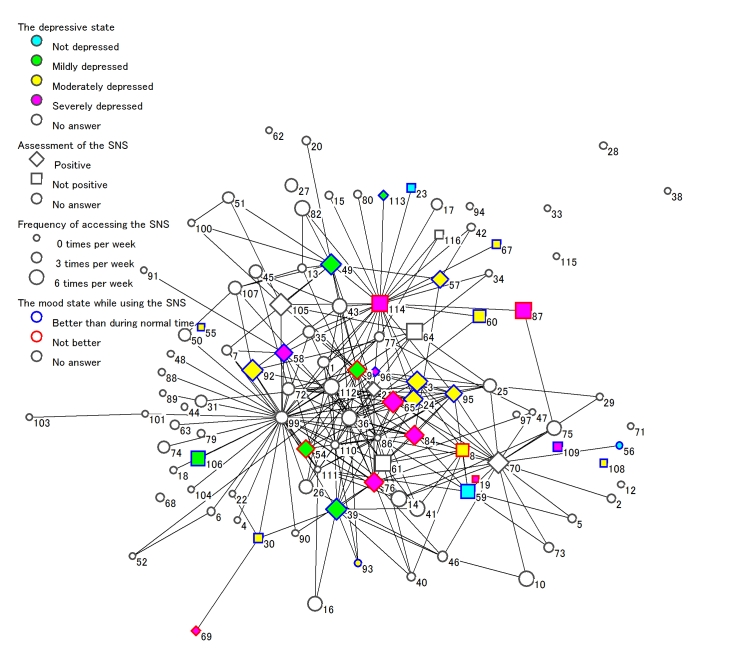
Social network in the SNS. Each node with a number represents one person. Numbers refer to anonymous registrants’ IDs, corresponding to Table 3. Each line between nodes indicates a “friend” relationship. Depressive states, assessment of the SNS, and mood states are explained in Table 2. We compared the mood states in two situations: (A) during normal time (time not using the SNS or Internet) and (C) while using the SNS. “Better than during normal time” means that the mood state in (C) is better than the mood state in (A).

Figure 3 depicts the social network of the 105 participants.

The number of cliques was 115, and the two biggest cliques each included five people, (IDs 9, 61, 76, 99, 112 and IDs 58, 86, 99, 110, 111), which meant they were friends with each other. The top five registrants (in order) as measured by degree centrality were 99, 114, 9, 70, and 36; by closeness centrality, were 99, 114, 9, 65, and 21; and by betweenness centrality, were 99, 114, 70, 9, and 49. People using the SNS frequently, having more friends, and with a “positive” assessment seem to be clustered around the central region of Figure 3. Some gatekeepers, such as 99, 114, and 70, who had high betweenness centrality, are also connected to individuals in the periphery of the figure. ID 114 had some friends who were moderately or severely depressed, such as 57, 58, 60, and 87. ID 114 was unsatisfied with <Infrequent usage>, although pointing out <Anonymity> and <Supporting each other> as advantages of the SNS. ID 87, who is a friend of 114, pointed out the potential harm, using the words “downward depressive spiral.”

In terms of the extent of interpersonal association, the odds ratio (95% confidence interval) for assessment of the SNS, depressive state, and frequency of accessing the SNS was 1.0 (0.5-2.2), 0.9 (0.3-2.6), and 0.9 (0.5-1.5), respectively.

## Discussion

### Main Findings

Most participants in this study were depressed, since 32/35 people (91%) could be diagnosed with mood disorders and 28/31 people (90%) had depressive tendencies, being either mildly, moderately, or severely depressed as measured by the depressive scale. The median age of participants was in the late 30s, older than users of MySpace or mixi, who tend to be adolescents. Almost half of valid respondents (16/34) were not working, which might suggest that people over 30 had trouble at work due to a depressive state. The data showed that the SNS was used by most participants since 34/37 (92%) had written a profile and 24/36 (67%) accessed the SNS three times or more per week. However, only a few people updated their blogs and/or registered in a large number of communities (see Table 1).

Qualitative content analysis indicated that the SNS could provide various types of peer support that users could select based on anonymity and ease of use. User-selectable peer support was passive, active, and/or interactive. On the other hand, there was the potential harm of a downward depressive spiral triggered by aggravated psychological burden. Social network analysis showed that users communicated one-on-one with each other or in small groups since the median of friends was three and the largest group included only five people. It also implied that a downward depressive spiral was related to friends who were moderately or severely depressed and friends with negative assessment of the SNS.

### Potential Benefits of SNS Participation: Peer Support

The SNS could provide user-selectable peer support. One participant felt encouraged, recognizing that a peer who was more depressed than he/she was continued to persevere (ID 9’s comment). At times, participants also felt empowered by giving support to others. As peer support was not a one-way interaction from the less depressed individual to the more depressed individual, it could be passive, active, and/or interactive. Accordingly, peer support might spread incrementally in the SNS. Moreover, participants could decide for themselves how many friends they connected with and how close they became to these friends. Therefore, a peer support SNS could meet participants’ needs, such as avoiding face-to-face communication and maintaining privacy, while providing peer support. Given that SNSs are widely used among the public, this suggests that SNSs offer effective opportunities for people with depressive tendencies to obtain peer support.

### Potential Harm of SNS Participation: Downward Depressive Spiral

As mentioned in the qualitative content analysis, a detrimental effect can result given that depressive tendencies can be exacerbated by cyber communication and dependency can be intensified for people with higher depressive tendencies. Dennis [6] also points out that an adverse outcome from peer support is the potential for emotional over-involvement that results in contagion stress. The downward depressive spiral emerged in part of the SNS, possibly because people with depressive tendencies tend to have greater dependency or because the mood state while using the SNS can worsen compared to normal time or while using the Internet if people had a “not positive” assessment of the SNS. In contrast, social network analysis showed that people with similar attributes, such as assessment of the SNS, depressive state, and frequency of accessing the SNS, did not tend to be connected with each other as a whole.

There is a potential discrepancy between the results of the qualitative content analysis and social network analysis. We interpret this discrepancy as follows. The SNS could, as a whole, prevent participants from experiencing the downward depressive spiral because it provides a domain where participants can face each other honestly. However, content analysis presented the possibility that the downward depressive spiral is an adverse event resulting from SNS participation. Future studies will be required to fully resolve this discrepancy.

### Limitations and Strengths

Our study has several limitations and strengths. We acknowledge that group members were not selected through theoretical sampling and were by no means a representative sample of patients with clinical depression. However, they were people with complaints of depression who used the SNS. A study reported that the suicide rate from 2003 to 2004 in the United States increased, and the influence of Internet social networks was included as a potential factor to consider [38]. The need to examine relationships between Internet social networks and depression or suicide must be addressed.

We also acknowledge that the sample size was very small (105 total group members). A limitation of this study is determining if the results are representative because the study included just one SNS, which had a small number of members. Moreover, the survey was done through the Internet, and the ratio of respondents was relatively low (34%). To address selection bias, we compared valid respondents with invalid/nonrespondents using SNS log files, which showed that people using the SNS frequently were selected. To address information bias, we confirmed the answers’ reliability and that the questionnaire responses corresponded with the SNS log files.

Finally, this study was designed as a cross-sectional survey, so we could not estimate causality between the SNS’s effects and outcomes. As new information technology on the Internet is being developed, recently referred to as Web 3.0 [39], new technologies and services emerge and are put to use even before their benefits or harms are assessed. It might be not practical to examine the SNS by an experimental study because it will not be used until a few years later. However, it is useful to research existing technologies using observational studies since most new technologies stand on the shoulders of existing technologies.

### Implications

We believe that this study has three public health implications. First, this study evaluated potential benefits and harm of SNSs, which are widely used among the public. Since SNSs can address individual needs of the public, it is important to analyze consumers’ needs from the viewpoint of consumer health informatics [40,41]. Finding a balance between potential benefits and harms [15] contributes to both health and eHealth providers as well as patients and SNS users. Moreover, these benefits and harms could spread as “network phenomena” [18-20]. From the perspective of public health, it is important to prevent initial undesirable events. Desirable intervention would also provide desirable results.

Second, this study used mixed methods, combining content analysis of qualitative data with social network analysis of quantitative data. These data were derived from questionnaires and SNS log files. A qualitative approach is required to analyze relationships among people, and therefore this mixed methods strategy could be useful for exploring social networks.

Finally, this study suggested that the SNS was a kind of social network, as depicted in Figure 3. Features of Web 2.0 in health care, called Medicine 2.0, are social networking, collaboration, participation, apomediation, and openness [42]. Therefore, in the future, personally controlled online health data using Google Health or Microsoft HealthVault [43] could be linked by a common application programming interface for social applications across multiple websites (eg, OpenSocial) [44]. In future studies we believe it will be necessary to consider the influence of interpersonal associations or social networks.

### Conclusions

A peer support SNS for people with depression might offer effective opportunities to obtain support for people with depressive tendencies because the SNS can provide user-selectable passive, active, and/or interactive peer support based on anonymity or ease of use. It can meet participants’ needs, such as avoiding face-to-face communication and maintaining privacy, while providing peer support. On the other hand, to avoid a downward depressive spiral, we recommend that participants refrain from using the SNS when they feel that the SNS is not user-selectable (eg, when they get egocentric comments, when friends have a negative assessment of the SNS, or when they have an additional psychological burden).

As communication on the Internet becomes more social, the mixed methods strategy used here, combining content analysis of qualitative data and social network analysis of quantitative data, is available to explore benefits and harms of this communication.

## Multimedia Appendix 1

Glossary of terms used in this article

## Multimedia Appendix 2

Framework of analytic methodology

## Abbreviations

SNS: social network service
