# Supplementary material for: Potential Benefits and Harms of a Peer Support Social Network Service on the Internet for People With Depressive Tendencies: Qualitative Content Analysis and Social Network Analysis
Source: J Med Internet Res. 2009 Jul 23;11(3):e29. doi: 10.2196/jmir.1142 (PMC2762850; doi:10.2196/jmir.1142)
Supplement: Supplementary file 1 [file jmir_v11i3e29_app1.pdf]

Takahashi Y, Uchida C, Miyaki K, Sakai M, Shimbo T, Nakayama T
Potential Benefits and Harms of a Peer Support Social Network Service on the Internet for People With Depressive Tendencies: Qualitative Content Analysis and Social Network Analysis
J Med Internet Res 2009;11(3):e29
URL: http://www.jmir.org/2009/3/e29/
doi: 10.2196/jmir.1142

Mutlimedia Appendix 1: Glossary of terms used in the article

Social Network Service (SNS)	A SNS is a Web 2.0 application that is an Internet-based service for building online social networks to allow individuals to share interests and activities, e.g. MySpace or Facebook.
“Friends”	“Friends” are online friends whose profiles are featured as links on one’s own profile in the SNS.
“Communities”	“Communities” are online communities for people with similar interests or activities in the SNS.
Measures used in the study	
Zung Self-rating Depression Scale	The depression scale is a validated self-rating scale to assess depressive state. It ranges from 20 (no depression) to 80 (major depression), and was categorized as follows: not depression (-39), mild depression (40-47), moderate depression (48-55), and severe depression (56-)
Numerical rating scale	The numerical scale ranged from 0 (no pain) to 10 (worst possible pain), which is usually used to assess present pain intensity. In this study, it ranged from 0 (good mood) to 10 (bad mood), which was used to measure mood state in the following three situations: (1) during normal time (time not using the SNS or the Internet), (2) while using the Internet, and (3) while using the SNS.
Positive assessment / not positive assessment	Items related to outcomes in the assessment of SNSs were evaluated by the question, “Do you think you became more positive to depression compared to before participating in the SNS?” Response choices were: “much more positive,” “more positive,” “no change,” or “more negative.” “Much more positive” and “more positive” were classified as “positive assessment”, and the other responses were classified as “not-positive assessment.”
Social network analysis	Social network analysis is the study of social structure that provides a means to quantitatively explore social relationships between people. Commonly used by sociologists, its use in health-related fields is increasing as an effective approach for research centered on describing, exploring, and understanding relational aspects of health.
Social network	A social network is a social structure made of individuals who are linked together by

	social relationships. In this study, the structure of participants in the SNS who are linked together by the relationship of “friends” was regarded as a social network.
Centrality	Centrality is an index of importance or prominence of individuals in a social network. There are several types of centrality: degree centrality, closeness centrality, and betweenness centrality.
Degree	Degree is the sum of individuals who are linked together; people with a higher degree centrality have more “friends.”
Closeness	Closeness is the distance between individuals; people with a higher closeness centrality are able to reach others more easily or directly.
Betweenness	Betweenness is the number of times an individual connects pairs of others; people with high betweenness centrality are able to play a gatekeeper role, controlling the flow of resources in the network.
Clique	A clique is a group which included three or more individuals connected by all possible connections. All people in a clique directly connected to one another as “friends.”
Mixed methods	Mixed methods research combines elements from both quantitative and qualitative paradigms to produce converging findings in the context of complex research questions. In this study, “mixed methods” included statistical analysis, qualitative content analysis and social network analysis.
Qualitative method	
Grounded theory	Grounded theory is a systematic qualitative research method to generate theories regarding social phenomena, which was developed by Glaser and Strauss.
Concepts	Concepts are collections of codes of similar content that allows the data to be grouped.
Categories	Categories are broad groups of similar concepts that are used to generate a story line or a theory.
