# Supplementary material for: Potential Benefits and Harms of a Peer Support Social Network Service on the Internet for People With Depressive Tendencies: Qualitative Content Analysis and Social Network Analysis
Source: J Med Internet Res. 2009 Jul 23;11(3):e29. doi: 10.2196/jmir.1142 (PMC2762850; doi:10.2196/jmir.1142)
Supplement: Supplementary file 2 [file jmir_v11i3e29_app2.pdf]

Multimedia Appendix 2: Framework of analytic methodology

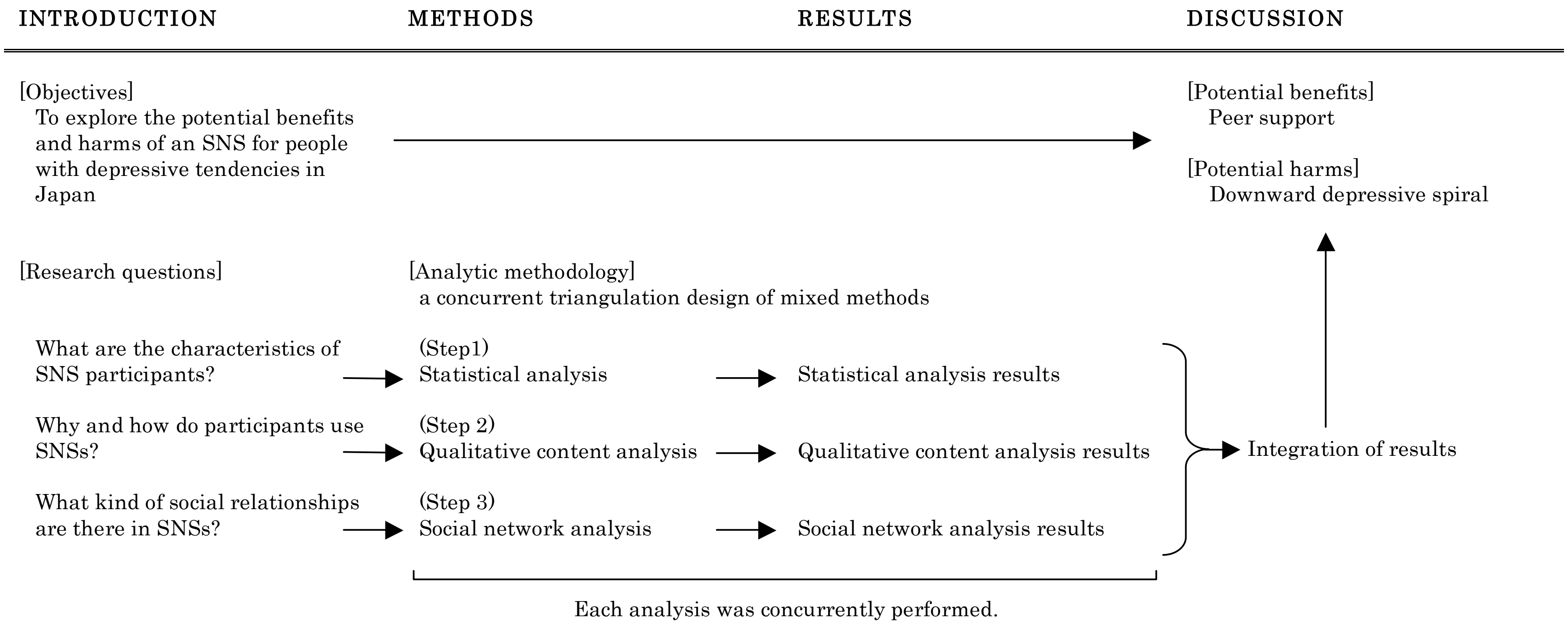

Takahashi Y, Uchida C, Miyaki K, Sakai M, Shimbo T, Nakayama T
Potential Benefits and Harms of a Peer Support Social Network Service on the Internet for People With Depressive Tendencies: Qualitative Content Analysis and Social Network Analysis
J Med Internet Res 2009;11(3):e29
URL: http://www.jmir.org/2009/3/e29/
doi: 10.2196/jmir.1142
